# The Effect of Traumatic Brain Injury on Memory

**DOI:** 10.21315/mjms2024.31.3.4

**Published:** 2024-06-27

**Authors:** Luqmanul Hakim Abdul Razak, Tedd Denis, Yoghaanjaly Murugiah, Weng Kei Yoong, Zamzuri Idris, Mohd Harizal Senik

**Affiliations:** 1 Department of Neurosciences, School of Medical Sciences, Universiti Sains Malaysia, Kelantan, Malaysia; 2 Brain and Behaviour Cluster, School of Medical Sciences, Universiti Sains Malaysia, Kelantan, Malaysia

**Keywords:** traumatic brain injury, short-term memory, working memory, long-term memory, virtual reality

## Abstract

Having a good memory is essential for carrying out daily tasks. People cannot study, plan, remember or navigate life effectively if they are memoryless. People may be at risk when mistakes made in the past will be repeated and lessons regarding danger cannot be learned. In the community, traumatic brain injury (TBI) is common and individuals with TBI frequently have memory problems. It is crucial to study how TBI affects memory to better understand the underlying mechanism and to tailor rehabilitation for patients with a range of pathologies and severity levels. Thus, this paper aimed to review studies related to TBI’s effect on memory. This review examined recent studies to learn more regarding and comprehend the connection between TBI and memory, including short-term memory (STM), working memory (WM) and long-term memory (LTM). This will undoubtedly have a big impact on how memory problems that may arise after TBI will be addressed. Virtual reality and other technological advancements have given the medical community a new way to investigate rehabilitative therapy.

## Introduction

The human brain is a fragile biological entity that is susceptible to many forms of illnesses and external or internal disorders. When a concern is related to external physical forces toward the brain (frequently observed in the case of blunt trauma, fall or mechanical accident), brain function disturbances across many aspects of cognition could occur (1). This serious medical condition is known as traumatic brain injury (TBI), which is one of the most debilitating neurological diseases in the world across all ages, accounting for more than 50 million sufferers each year (2). Along with the unfortunate medical consequences, the healthcare cost of burden for TBI is high, which was $200 million in the United States alone, contributing to economic setbacks in certain low-income countries such as Ethiopia (3).

**Figure 1:**
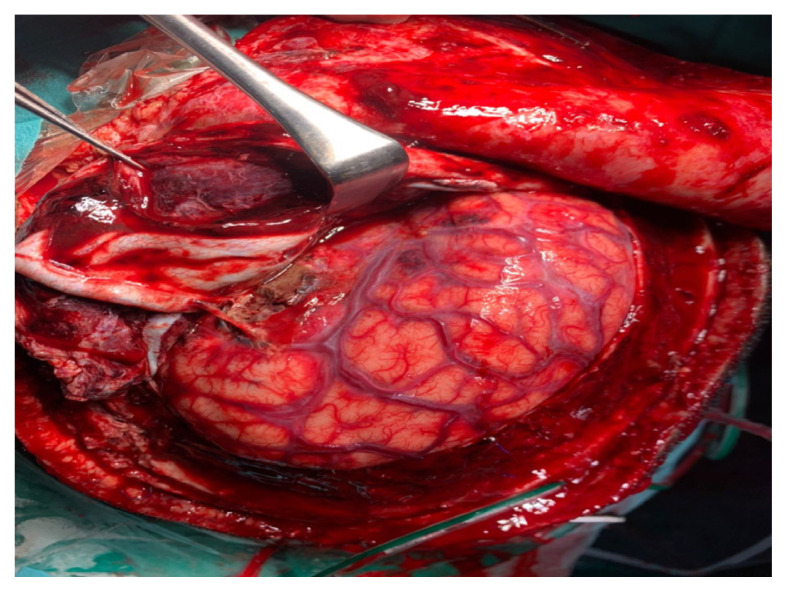
Brain with closed-head trauma

There are three categorisations of TBI depending on the physical mechanisms of brain injuries: the closed-head, penetrating and explosive blast TBIs (4). TBI could also be categorised in terms of its severity using the validated Glasgow Coma Scale (GCS) into mild (score 14–15), moderate (score 9–13) and severe (score 3–8) TBIs (5, 6). The first category of TBI, that is, closed-head trauma, is caused by nonpenetrating physical impacts on the brain, such as motor vehicle accidents, falls or rough sports activities. Figure 1 shows an example of a patient with closed-head trauma who had undergone surgery in the Department of Neuroscience of our institution with obvious brain contusion, traumatic subarachnoid haemorrhage and brain swelling. Most closed-head traumas are categorised as mild forms of TBI (7). Nevertheless, the mild physical impacts on the brain could cause significant neurological effects, manifesting symptoms such as a change in mental status (confusion and disorientation), loss of consciousness for up to 30 min, post-traumatic amnesia, memory deficit for up to 24 h and other neurological deficits that may or may not be transient (8).

**Figure 2:**
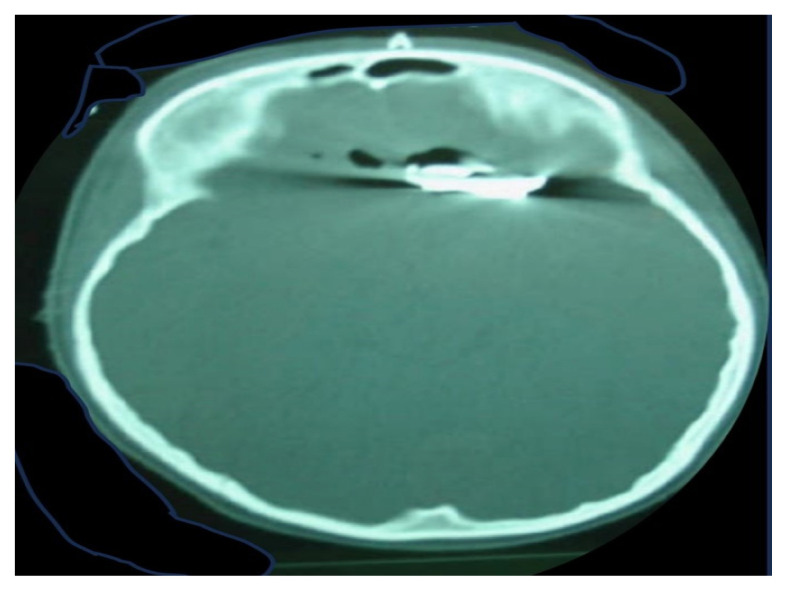
An image of a CT scan that indicates an iron piece penetration can be seen from the inside of the cranium

The second category of TBI occurs because of penetrating injuries toward the skull through the dura layer into the brain parenchyma (4). It is one of the most lethal forms of TBI, often occurring because of weapon-related assaults, such as knife stabbing or pistol shots, which are frequently observed in military combats, accidents or criminal attacks (9). One case where a foreign body (iron piece) penetrated the skull of a boy when he stood near his father while his father was repairing their car has been referred to our department. A computed tomography (CT) scan image of the child’s skull that indicates iron piece penetration observed from the inside of the cranium is displayed in Figure 2. The extent of the injuries in this form of TBI depends on various factors, such as the type and size of penetrating materials, the speed and kinetics of penetration and the areas of the brain injured (10). In most cases of penetrating TBI, surgical intervention is the treatment of choice to remove the object(s) of insult, promoting brain recovery. A significant number of patients would survive following surgery, although continuous cognitive rehabilitation would be required (11).

The third and relatively newer and unique category of TBI is the explosive blast TBI, experienced typically by military personnel in combat-related situations (12). It could be argued that explosive blast TBI was first conceptualised during the Global War on Terror, specifically during the Operation Enduring Freedom in Afghanistan and Operation Iraqi Freedom. During this time, it was found that there was a high incidence of individuals developing TBI due to the explosive blasts prevalent within the war zones (13). An explosive blast wave is produced by either the detonation of high explosives or the deflagration of low explosives under certain restrained conditions. In a high explosive blast, for example, rapid and sudden expanding hot gases are created by the explosive compound, causing a rapid and sudden increase in air pressure. Immediately after the explosion, the pressure drops suddenly and the compressed gases continue to compress the surrounding air, producing an intense blast wind that travels far from the initial site of the explosion (14, 13).

Whenever an individual is exposed to an explosive blast, the rapid shifts in the air pressure could injure the brain upon contact, leading to concussion and contusion. Apart from this, air emboli could be formed in the blood vessels and could increase the risk of cerebral infarction (15). Despite the clinical similarities of explosive blast TBI with the other two types of TBI, namely, closed head and penetrating TBIs, there are some distinct features. For example, patients with explosive blast TBI could be presented with early cerebral oedema and prolonged cerebral vasospasm, along with different diffuse axonal injuries (DAIs), compared with the other two types of TBI. Therefore, these features reserve the right for explosive blast TBI to be categorised as a unique form of TBI, apart from closed head and penetrating TBIs (13).

All of these causal factors of TBI could produce physical manifestations with different severity levels. To reliably determine the extent of brain injuries and their effects on a patient’s consciousness level, clinicians rely on the GCS. This scale requires an evaluation of the patient’s best eye, motor and verbal responses, which are then translated into corresponding numerical scores. The scores from these three responses are then added and the total determines the severity of the brain injury (16). The GCS is extensively utilised and validated for its prognostic indicators for brain traumas. Apart from this, the scale can be reliably used to monitor the deterioration or recovery of patients with TBI (6). The scoring system of GCS is illustrated further in Table 1.

**Figure 3:**
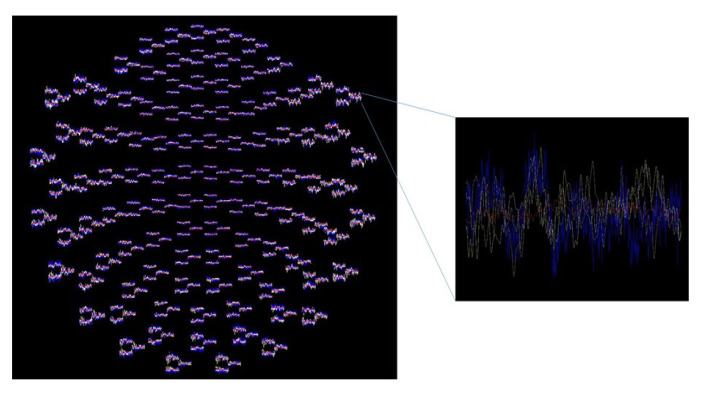
MEG data from a patient with a moderate head injury was examined at different memory tasks (red at rest, yellow, blue and white are three separate memory tasks)

Memory is one of the core cognitive domains that is known to be affected by all types of TBI. According to Zlotnik and Vansintjan (19), memory is a cognitive ability that allows the brain to encode, store, and recall information. This crucial cognitive function is controlled by several different brain regions, particularly by regions such as the hippocampus and the parietal and frontal lobes (20–23). Brainwaves are commonly used to study cognition, including memory (24). Our magnetoencephalography (MEG) data from a patient with a moderate head injury examined at different memory task levels are displayed in Figure 3. Depending on the different forms of memory categorisation, which will be covered in this mini-review, the clinical symptom manifestations and the brain areas damaged by TBI may be different or similar. Aside from that, the status of the memory rehabilitation therapy for patients with TBI employing virtual reality (VR) will also be reviewed.

### Traumatic Brain Injury Effects on Short-Term Memory

Short- and long-term brain damage, cognitive impairment with or without structural change, motor impairments, emotional problems, and mortality in both children and adults are among the major neurological abnormalities in the brain caused by TBI (25). Damage in the brain induced by TBI could also extend to the parts governing the ability to utilise, manipulate and retain short-term memory (STM).

According to Postle and Pasternak (26), STM refers to the active retention of knowledge while it is not available from the surroundings. It refers to some memory systems that are involved in the retention of information (memory chunks) for a small amount of time, frequently up to 30 s. It is also known as primary memory, short-term storage or active memory (27). STM is the ability to store a small quantity of information in the mind and to keep it accessible for a short period (28). It is very brief in that it only lasts for a few seconds if it is not rehearsed or actively maintained (29). In addition, STM is limited, whereby it can hold only seven items at once, plus or minus two. For instance, according to a study by Cowan (30), in STM tasks, people can remember approximately seven chunks. Thus, STM is necessary for everyday functioning, and it can be frustrating and even debilitating to experience STM loss.

A study by Malojcic and colleagues (31) examined how STM and attention are affected by mild TBI (mTBI). A series of computerised tests assessed STM decision time, basic response time and sustained visual attention. People who had mTBI had performance issues, with prolonged visual attention, STM scanning, and a tendency to make slower decision-making. It is postulated that poor central information processing is the cause of these observed alterations in the cognitive performance of patients with mTBI. According to the findings, mTBI can cause cognitive deficits that last for several months after the injury.

The STM function involves the dynamic interactions of various structures of the brain, such as the cerebral cortex, hippocampus, parahippocampal gyri and thalamus (32–35). Hence, a TBI event that inflicts damage on these structures would in effect lead to STM memory deficit. A particularly important brain area is the prefrontal cortex (PFC), which has been linked to memory function. According to Warden and Miller (36), PFC is crucial for flexible, context-dependent behavioural regulation, which is essential for maintaining STM. In STM, the dorsolateral PFC (DLPFC) is a substantial neural structure (37). It is generally known that the DLPFC, which is crucial for both memory and attention functions, activates a large number of neurons during the performance of these tasks (38). Cho and colleagues (37), for instance, investigated the relationship between STM impairment and DLPFC injury in patients with TBI using diffusion tensor tractography. A total of 42 healthy control participants and 46 chronic patients with mild TBI were enlisted. For both hemispheres, the prefrontal-thalamic tracts’ fractional anisotropy and fibre number were determined. The findings demonstrated that in individuals with mTBI, STM impairment was strongly related to DLPFC damage.

A wide range of issues, such as emotional, behavioural and physical issues, as well as substance use disorders, physical ailments, prescription and self-administered medicines, and symptom exaggeration, are included in the differential diagnosis for posttraumatic cognitive impairments. Treatments primarily aimed at cognitive impairments require a thorough neuropsychiatric examination for such issues (39). Non-pharmacological therapies such as education, realistic expectation setting, cognitive rehabilitation, environmental changes and lifestyle changes are first-line treatments for post-traumatic cognitive impairments (39). Changes in the lifestyle of a patient with TBI, for example, involve measures such as physical exercises and sufficient rest (40). Apart from these activities, diet and vitamin supplementations are also associated with the reduction in TBI memory deficit. According to a review by Javaid et al. (41), studies on both humans and animals revealed that diets that include choline or choline-derived foods could decrease inflammation, boost neuroprotection and enhance memory.

Cognitive rehabilitation, a professional specialty that includes multidisciplinary work aimed at the recovery and compensation of cognitive functions altered by cerebral damage, is essential for people with TBI (42). A cognitive rehabilitation programme can help someone regain his or her capacity for processing, interpreting and responding effectively to external information. For instance, a case study by George (43) on the outcomes of cognitive-communication intervention in TBI demonstrated the cognitive-communicative problems caused by TBI in a female adult from India and the intervention outcomes. A 43-year-old individual participated in 20 sessions of a cognitive-communication intervention that used a domain-general adaptive training paradigm and tasks that were applied to regular cognitive-communication skills. After the intervention, the participant’s perception, STM and working memory (WM) all showed improvements and perseverations and naming challenges were reduced. George (43) concluded that through the right selection of objectives and activities pertinent to each individual’s functional requirements, rehabilitation of individuals with moderate-to-severe brain injuries may be accomplished efficiently.

### TBI Effects on WM

It is often easy to get confused by the concept behind the terms WM and STM. STM is withholding small amounts of information for a short period to act (44), whereas WM is manipulating the stored information to put on the, as the name suggests, ‘working table’ to perform complex tasks (45). Remembering a phone number that you just learned to stay long enough to dial on the phone involves STM, whereas solving a given mathematics question involves WM. WM involves encoding information, maintaining information in conscious awareness and retrieving information to perform executive control over information (46, 47), and it involves subcomponents such as central executive control, the visual-spatial sketchpad and the phonological loop (48).

Executive functions are very important in our daily life, which could be observed in driving (focus on the driving task while filtering information, adapting to the road conditions, etc.) (49), and they include inhibition, cognitive flexibility and WM. WM involves higher cognitive functions such as decision-making (50), mathematics-solving ability (51) and reasoning (52). Impairment in WM could pose difficulty in a range of executive functions and could affect our quality of daily living life. Several neural populations are activated while exercising WM, such as the PFC, basal ganglia, thalamus and brainstem (45), and frontoparietal regions (53). Furthermore, increased gamma-band oscillations were observed during the maintenance of information (54, 55). However, the increased interhemispheric gamma connectivity observed during information maintenance in patients with TBI and patients with TBI and major depressive disorder might suggest dysfunction due to DAI (54). DAI is the damage of axons in the brain that is often observed in blunted-head injury (56), and the acceleration and deceleration of the brain in the skull could contribute to the occurrence of blunt-force head trauma (57).

A study by Taing and his colleagues (58) suggested that the activation of the left occipital gyrus increased during the encoding of information and the activation of the bilateral cerebellum and left calcarine sulcus decreased when patients with TBI try to maintain high loads of information. Functional magnetic resonance imaging (fMRI) and Sternberg delayed match-to-sample were used to examine the brain regions that were activated while performing tasks in different subprocesses such as encoding information, maintaining information, and retrieving information. Compared with the healthy control participants, the activation in the right DLPFC was decreased in patients with TBI during the WM maintenance phase (58). The DLPFC plays an important role in the executive control of WM (59). The Sternberg task was developed by Sternberg in 1966. It has been used in several studies to study WM as it allows researchers to measure separate stages of encoding, maintaining, and retrieving, which correspond to the WM subcomponents (60–62). In a classic Sternberg task, participants will start with memorising (encoding) a set of digits or letters shown, followed by a maintenance phase where participants will be looking at a blank screen for a few seconds before entering the retrieval phase. In the retrieval phase, participants have to decide whether the stimuli shown on the screen appear in the encoding stage memory set (60). Low- and high-cognitive-load conditions are given to participants. Two (58) or three (60) list lengths are given as low cognitive loads and six (58, 60) or eight (63) list lengths are given as high cognitive loads. Furthermore, the Sternberg task can be conducted in a visual or auditory design (60). Taing and his colleagues (58) recruited patients with moderate-severe TBI with a mean of 2 months post-injury, a mean of 23 months post-injury and healthy participants as control, and they suggested that patients with TBI and WM impairments pose difficulty in modulating brain activity compared to typical healthy individuals during the WM encoding and maintenance phase.

Recovery from TBI does not ensure full recovery in WM. Difficulties or impairment in WM performance is observed to continually extend in patients with TBI even after TBI recovery (64). Gorman and his colleague (65) examined the relationship between WM development and the age at which a TBI was acquired as well as the degree of the injury. In the study, patients with severe TBI and mild or moderate TBI acquired TBI at the age of 6 years old to 15 years old, and participants with orthopedic injuries were recruited. Category listening span task and visuospatial span were used to measure verbal and visuospatial WM in this study. It was a longitudinal study that could provide more insight into the development of WM in pediatric patients with TBI and the cognitive abilities were assessed 2, 6, 12 and 24 months after TBI. The results of the study suggested that children who acquire severe TBI at an early age have a slower rate of development compared to the other groups, but improvement continued to be observed after 1 year to 2 years post-injury, which was in contrast with the study by Levin and his colleague in 2002 (66), who observed that WM performance declines in children who acquire severe TBI at a younger age. A longer follow-up on post-injury in children was suggested to provide appropriate interventions earlier to improve the possible behavioral outcome of the children. Future research combining neuroimaging techniques and the longitudinal study was also suggested by the researchers to gain more understanding of the neurological mechanisms that were involved in the development of WM after acquiring TBI.

### Traumatic Brain Injury Effects on Long-Term Memory

Long-term memory (LTM) is associated with the individual cognitive ability to store information for a long time or indefinitely, and it can be categorised into two different categories: implicit and explicit memories. Both types of memory are differentiated in terms of conscious recollection. Implicit memory does not require conscious effort to retrieve, such as riding a bike and having a conversation (67). Conversely, explicit memory is a form of memory that requires conscious effort, such as facts or event recollection (68). Implicit memory could further be categorised into four types: i) procedural, ii) associative, iii) non-associative and iv) priming memories. Meanwhile, explicit memory is further subdivided into episodic and semantic memories. Owing to the myriad subdivisions under LTM and the diffuse nature of TBIs, it is difficult to predict the effect of TBI on this type of memory (69). For instance, semantic LTM memory might be affected by a particular TBI event, whereas other subtypes such as episodic memory might be preserved in similar conditions (46, 70). Despite this difficulty, the various studies that are highlighted in Table 2 recognise that TBI certainly induces significant changes in the performance of LTM, regardless of the subtypes that might be affected.

#### Effect of Traumatic Brain Injury on Long-Term Memory

It is difficult to view the effect of TBI on LTM as a whole because LTM function is not categorised as a single unit (28). Thus, this paper collected several articles related to the effect of TBI on LTM. The collected articles are summarised in Table 2.

### Semantic Organisation to Traumatic Brain Injury

Semantic memory allows an individual to understand the meanings, symbols and conceptual facts regarding the world, and TBI episodes could impede this crucial functioning of the brain (84). Research investigations have been widely conducted to investigate the TBI effects on semantic memory (85–87). One of the earliest studies in the 1990s was conducted by Goldstein et al. (70), who concurred that semantic (categorical) encoding of words results in greater recognition and cued recall in patients with TBI, albeit to a lesser extent than that in controls. This finding was consistent with the later research by Vakil and colleagues (71), where patients with TBI did not exhibit differential delayed recall based on the significant information in the story presented by the researcher. Hence, patients exhibited better retention of the more important information units. Interestingly, the results from the patients did not differ from those from the control group. This leads to the conclusion that patients with TBI often struggle with other memory functions, such as retrieving important information, rather than the semantic understanding of the information itself (71).

In 2000, Perri and colleagues (72) found a contradictory result from previous studies, where they found a decreased capacity to learn and memorise semantic knowledge. For instance, during word-list memory tests and when asked to respond to questions on general knowledge, these individuals showed a diminished capacity to spontaneously use semantic knowledge. Semantic memory and the organisation of semantic knowledge during the early stage of recovery from TBI were also discovered in the study by McWilliams and Schmitter-Edgecombe (73). They recruited 24 participants with moderate-to-severe TBI and 24 controls. Both groups were asked to describe three living and three nonliving objects as if they were describing them to someone who had never heard of or seen such things before. The TBI group produced object definitions that conveyed the main idea and contained information regarding superordinate categories less frequently than the control group did. The proportion of physical specific features produced by the TBI group was likewise lower, and the creation of fewer physical specific features was correlated with the poorer production of the core concept. Despite these discrepancies between the groups, both groups produced more associative and physically specific characteristics for nonliving objects and more physically specific features for living objects than general feature information. Hence, the findings are similar to those of the previous study by Perri et al. (72), suggesting decreased efficiency in the ability to access semantic information following moderate-to-severe TBI, which influenced core concept production, despite the intact organisation of semantic knowledge.

### Effect of Traumatic Brain Injury on Episodic Memory

Endel Tulving first coined the term ‘episodic memory’ in 1972 to describe an individual’s capacity to remember specific prior events, including where and when they occurred (88). In contrast to other types of memory, episodic memory is specifically situated in the past and accompanied by the experience of remembering, on the other hand, the knowledge that the person acquired is just factual and devoid of any personal history.

Episodic memory deficit is observed in patients with mTBI, where it has been reported that they have difficulty in accessing episodic memory tasks such as immediate recall, delayed recall and recognition (74). The current research has moved its focus toward examining the affected area of the brain of a patient with TBI and episodic memory. Fortier-Lebel and colleagues (75) carried out two experiments to examine the effects of a single, acute mTBI on the structural alterations in the brain and episodic memory. In the first experiment, they compared 52 patients with TBI to 54 healthy controls, evaluating verbal episodic memory using a word recall test for 6 months. In the second experiment, they used magnetic resonance imaging (MRI) to estimate the hippocampus volume in a subgroup of 40 participants from Experiment 1 (20 patients with mTBI and 20 controls) and found that there were positive correlations between memory performance scores and hippocampal volume.

Alternatively, Taing and colleagues (58) pointed out that the temporal lobe is significant for encoding and retrieving episodic memories. The researcher investigated the connection between episodic stimulus encoding and temporal lobe activity using fMRI. During an fMRI run, participants encoded face, scene and animal stimuli. Participants had to properly identify previously shown stimuli across two presentation runs in an out-of-scanner task (each in-scanner stimulus was presented twice). A total of 43 patients with moderate–severe TBI were included and their characteristics were compared to those of 38 healthy controls. In the first presentation, but not the second, the TBI group displayed worse episodic memory for faces and scenes. Behaviour deficits were only found for faces when episodic memory across all presentation runs was analysed. Interestingly, the only between-group difference in face processing was observed on fMRI when patients with TBI had an elevated signal in the middle temporal gyrus that extended to the superior temporal sulcus. These results support the hypothesis that after TBI, episodic memory is particularly impaired for complex stimuli such as faces and that strong behavioural inefficiencies are reflected in enhanced activation in particular temporal lobe areas during encoding. Depending on how the stimuli were presented, different groups behaved in different ways.

### Effect of Traumatic Brain Injury on Priming Memory

Another part of memory that is often associated with individuals with TBI is priming memory—which is a part of implicit memory that it is responsible for activating an association or representation in memory prior to a stimulus or task being presented. There is very limited current research available regarding priming memory and patients with TBI. Initially, Vakil and Sigal (88) examined the same group of patients with TBI on perceptual priming (i.e. partial-word identification) and conceptual priming (i.e. category creation) tasks (i.e. free recall). On the perceptual priming test, the groups did not substantially vary from one another. However, on the conceptual priming task and the declarative task, patients outperformed controls. This separation of perceptual and conceptual priming is somewhat supported by future research on patients with TBI. For instance, Watt et al. (76) found that individuals with TBI had retained priming when tested using the word stem completion task under the full attention condition. Patients under the divided attention condition, in contrast to controls, displayed a diminished priming effect. Although the word stem completion task is an implicit memory task, the authors’ interpretation of their findings suggests that it still demands attentional resources that are depleted after TBI.

Vakil and Oded (77) recruited 40 participants, where 20 volunteers were grouped under control and another 20 patients with TBI were under another group. Both a ‘saving’ task and a ‘priming’ task were administered to each participant. Each of these activities was supplemented with a cued recall task. Each of the individuals underwent three testing sessions. The saving task was the focus of two of these sessions that were spaced out by two weeks. A different session was used to give the priming task. The priming exercise was given to half of the participants one week before the two saving sessions. The priming exercise was given to the other half of the participants one week after the second saving session. Based on the research, they found that implicit memory (i.e. word stem completion) is preserved in patients following TBI only when based on the reactivation of pre-existing knowledge but not when dependent on forming new associations.

### Effect of Traumatic Brain Injury on Procedural Memory

The memory component known as procedural memory helps people recall the motor and executive abilities needed to complete a job. It is a system of executive control that directs action and typically operates on an unconscious level. Automated retrieval of procedural memory for use in carrying out difficult motor and cognitive skill-related operations is performed as needed. This helps individuals with everyday tasks, for example, riding a bike and tying a shoe.

Using procedural learning as an index of implicit memory, Ewert et al. (78) compared 16 adults with severe TBI to matched controls on a variety of implicit memory tasks (such as mirror reading, maze learning and pursuit rotor tasks) and multiple explicit memory tasks (such as word recognition and a declarative memory questionnaire). They claimed that although those with TBI had difficulties with explicit memory tasks, they had improved throughout multiple sessions concerning all implicit memory activities.

Later research by Shum and his team (79) compared the performance of 16 persons with TBI with 16 matched controls on explicit (word fragment and general knowledge tasks) and implicit (graphic cued recall and semantic cued recall) memory tasks. They found that people with severe TBI performed noticeably worse on the two explicit memory tasks than they did on the two implicit memory tasks. These research findings suggest that implicit memory survives TBI in adults, in contrast to explicit memory.

In 2019, Rigon and colleagues (80) conducted a study to determine if patients with TBI are impaired in a task of procedural memory as a group and to determine if there are any discrepancies in performance across individuals. They recruited 36 individuals with moderate–severe TBI and 40 healthy comparisons (HCs) for the rotary pursuit task and then examined their rate of learning and their retention of learning. According to the studies, although the group of people with TBI spent much less time on tasks, they did not retain significantly less procedural learning, and overall, their rate of learning was comparable to that of HCs. However, there were significant individual variations in both groups, suggesting that some people might not benefit from therapeutic strategies that make use of a healthy procedural memory system.

### Effect of Traumatic Brain Injury on Learning

One of the big questions in the topic of learning in TBI is whether an individual would still be able to learn or acquire new skills after suffering from TBI. Mutter et al. (81) concurred that people with severe TBI showed reduced performance on the indirect measure of sequence learning but normal performance on the direct measure. Contradicting later research, patients with TBI were assessed using the serial reaction time (SRT) task by Vakil et al. (82), utilising explicit and implicit measures of sequence learning. The results imply that the patient group exhibits a distinctive pattern of learning impairing both the explicit and implicit measures of sequence learning.

One of the recent research projects conducted by Schwizer Ashkenazi et al. (83) recruited 26 individuals with TBI and 28 healthy controls to examine how TBI affected implicit sequence learning (ISL) using an eye-tracked variation of the common SRT paradigm. In addition to reaction time (RT), this ocular SRT (O-SRT) task allows the creation of accurate anticipations (CA) and stocks, reflecting other crucial ISL characteristics. On the basis of the RT, ISL shows a decrease in groups with TBI. However, the creation of CA showed enhanced learning with deficiencies in the stages of interference and recovery from interference. The researcher suggested that the high TBI group stuck rate could be linked to the TBI-related lack of initiative and/or conservative response bias, which is observed as the key factor contributing to ISL deficiencies. The TBI group, in contrast to controls, was unable to mobilise their comparatively unharmed spatial WM capacity to support their ISL performance (83).

#### VR-based Cognitive Rehabilitation Therapy for Traumatic Brain Injury-Induced Memory Deficit

Owing to the multiple cognitive domains that are also affected by the diffuse nature of TBI, the management options usually involve the recovery of the brain that is not exclusive to memory alone. Various studies have categorised different types of interventions for TBI, but most of these interventions, especially the ones that are related to therapy-based activity, could be broadly categorised under cognitive rehabilitation therapy.

Cognitive rehabilitation therapy is a set of interventions designed to improve an individual’s ability to perform cognitive activities following trauma and injuries by restoring past learned cognitive skills and applying compensatory skills to cater to the current cognitive status (90). Cognitive rehabilitation poses challenges to clinicians worldwide due to the absence of a solid theoretical base for evidence-based therapies and their monitoring parameters. One of the reasons for this challenge is partly due to the individual variation in brain responses following injuries. Even the same area of the brain that is injured in different patients produces diverse cognitive responses, which, in turn, may affect the cognitive recovery time of patients with TBI (91). Nevertheless, various evidence-based recommendations are available. Some of the approaches in memory rehabilitation in patients with TBI utilised word lists, paragraph hearing, visual imagery and linguistic approaches during the rehabilitation process (92).

In 2015, a multicentre, randomised Rehabilitation of Memory following Brain Injury (ReMemBrIn) trial was conducted to investigate the effect of a memory rehabilitation programme among 328 participants with and without brain injury. Unfortunately, this trial found no clinically beneficial outcome when it comes to using a memory rehabilitation programme (93, 94). Nevertheless, more studies are needed to establish this fact as rehabilitation interventions could vary among studies. In the ReMemBrIn trial, 10 group sessions were conducted once a week for 10 weeks. The participants underwent restitution strategies for memory retraining, compensation strategies and activities to enhance memory encoding and retrieval, among others. Meanwhile, another memory rehabilitation programme was conducted by Fleming et al. (95) among three patients, stretched only for 8 weeks of training and divided into eight sessions (1 h–2 h per session), with slightly different interventions. Different from the ReMemBrIn trial, this research found improvement in memory of subjects following TBI, although admittedly, the sample sizes were very small to generalise to a larger TBI population. Nevertheless, Das Nair et al. (94) reported that there was significant heterogeneity within the sample due to the broad criteria in the trial. Therefore, further research could identify the subgroup from the TBI population that would most likely benefit from the memory rehabilitation programme and could fine-tune the type of interventions in the cognitive rehabilitation therapy employed.

One of the advanced modalities used in cognitive rehabilitation therapy is VR in therapy sessions (96). Although VR-based cognitive rehabilitation therapy had indeed been discussed decades earlier, it has remained an area of research and clinical interest for its future potential to be widely used. For example, the current systematic reviews conducted by Barman et al. (92) and Despoti et al. (97), as well as the scoping review by (98), observed that immersive VR-based therapy had been associated with cognitive improvements across various domains, including memory in the TBI population. This is supported by various studies using VR, where patients demonstrated significant improvement in memory performance following TBI. The details of these studies are summarised in Table 3.

Despite the promising benefits of VR and the research enthusiasm accompanying it, there is still relatively little evidence to support the routine clinical use of VR-based therapy in improving cognition, particularly in preserving the memory function of patients with TBI (98). This is evident as most cognitive research utilising VR programmes consistently pointed out the small sample size of the participants and the short duration of therapy as the limitations in their studies (Table 3). Some of the reasons that this is the case are the negative outcomes found in trials such as the ReMemBrIn trial, the high cost of the VR apparatus and the heterogeneity of VR programmes (103), and the negative perception and skepticism among patients (especially in the elderly population) and therapists (102).

On the basis of the core limitations addressed by previous research, one of the practical approaches for future advances in VR-based memory rehabilitation therapy is to conduct a multiregional, randomised control trial involving large participants of specific TBI subtypes or populations. To achieve this ambitious research goal, however, a stepwise plan needs to be implemented as suggested by Brassel et al. (104) to ensure effective scale-up research operations and optimise the cost–benefit outcomes. According to the research team, this stepwise plan can be conducted in three main steps: i) the codesign steps, where the researchers design the VR approaches with feedback from the end-users (therapists and patients); ii) a feasibility study to assess whether the VR design can be applied in a larger population, and iii) a controlled trial, where large-scale randomised studies will be conducted to test the VR approach.

VR technology in rehabilitation should also utilise multimodal stimulatory activities rather than focus on a visually based system. It is known that VR technology is extensively used for the immersive video gaming experience, which recruits an individual’s visual, auditory and motor systems that are actively engaged during the gameplay. Because of this, some research saw potential in using VR gaming as a rehabilitation option in patients with TBI. For example, an fMRI study conducted by Caglio et al. (101) on a 24-year-old patient with TBI revealed increased activation in the hippocampal and parahippocampal areas, suggesting an improvement in memory functions after the VR gaming session. Meanwhile, a pilot study conducted by Ustinova et al. (105) among nine participants with TBI found considerable improvements in their motor aspects, such as postural stability, gait and upper extremity movements, signifying multiple potential benefits.

Lastly, a VR intervention could be designed in a suitable way to be combined with other forms of cognitive rehabilitation therapy. This approach is pointed out by the systematic review conducted by Cano Porras et al. (106) in assessing the use of VR during the rehabilitation of balance and gait. The review found additional benefits of using VR in combination with other forms of intervention such as neurodevelopmental treatment, transcranial direct current stimulation and robotic training. Extending this suggestion to memory rehabilitation following TBI can also extend this benefit, considering that different types of memories affected by TBI might react differently to the diversity of the intervention options available for use. Nevertheless, subsequent studies could be conducted to test this speculation.

## Conclusion

TBI causes memory impairment that manifests across a wide range of memory activities. The most severe cognitive effect of TBI is undoubtedly memory impairment, although there have been inconsistent findings on its effect across several cognitive domains. The information provides opportunities and relevance for current research to further study and understand the relationship between TBI and memory. This will certainly have significant implications for strategies to address approaches to memory difficulties following TBI. The advancement of technology such as VR has provided the healthcare profession with a new avenue to explore rehabilitation therapy. It has certainly changed the way health professionals conduct their therapy. For some, it might be controversial because of its nature but it undeniably has an effect toward patients with TBI. Therefore, further research is still needed to confirm and understand the effectiveness of the technology used in the therapy.

## Figures and Tables

**Table 1:** The classification of TBI severity using GCS scoring. Adapted from Mehta and Chinthapalli (16); Nair et al. (17) ‘with’ Teasdale and Jennett (18)

Domains of responses	Severity of responses	Score
Eye response	Spontaneously open	4
	Open after a verbal command	3
	Open to pain	2
	No response	1
Motor response	Able to follow command	6
	Able to localise pain	5
	Withdraw from pain	4
	Flexion response to pain	3
	Extension response to pain	2
	No response/No movement	1
Verbal response	Oriented	5
	Confused	4
	Inappropriate words	3
	Incomprehensible sounds	2
	No response	1

Notes: Total scores (TBI severity): 13–15 (mild), 9–12 (moderate), 3–8 (severe)

**Table 2:** The effect of TBI on LTM

LTM components	References	Objectives	Methodology	Conclusion
Semantic memory	Goldstein et al. (70)	To determine if semantically processed words have a memory advantage over other types in severe closed-head injury (CI) survivors (Ss).	Words remembering test with semantic (categorical), physical (letter), or auditory (rhyme) properties (*n* = 16 CI patients, 16 healthy controls; 20–49 years old).	Memory performance in CI Ss is improved when semantic characteristics are being focused on, despite requiring more mental effort.
	Vakil et al. (71)	To investigate the long-term memory recall ability in CI patients compared to control.	The Logical Memory subtest of the Wechsler Memory Scale (WMS) was administered (*n* = 40 CI patients, 40 Ss controls). The recall was evaluated 40 min and 24 h after test administration.	Based on the findings, CI patients struggle to selectively recall the most crucial information after a considerable delay.
	Perri et al. (72)	To determine whether the CI-induced semantic processing issue is caused by the disruption of access to an intact semantic system or knowledge loss from a degraded semantic storage.	Automatic semantic priming, picture naming, and semantic judgement tests were administered (*n* = 15 CI patients, 14 healthy controls; mean age = 22.3 years old).	CI patients automatically access semantic memory at a typical rate. However, their performance was reduced compared to the control in the naming and semantic judgment tests.
	McWilliams and Schmitter-Edgecombe (73)	To examine semantic memory and the organisation of semantic knowledge during the early stage of recovery from TBI.	Participants described three living and three non-living objects as if they were describing them to someone who had never heard of or seen such things before (*n* = 24 moderate-to-severe TBI patients, 24 healthy controls). The verbal definitions were examined at a feature level and for whether they communicated the core concept (i.e. could a blind later identify the object).	The findings suggested a decreased efficiency in the ability to access semantic information following moderate-to-severe TBI, which influenced core concept production, despite intact organisation of semantic knowledge.
Episodic memory	Konrad et al. (74)	To investigate the long-term cognitive and emotional sequelae of mild TBI.	Several neuropsychological tests were administered (*n* = 33 mild TBI patients, 33 healthy controls) including: i. Auditory Verbal Learning Test (AVLT) ii. Structured Clinical Interview for DSM-IV Axis 1 Disorders (SCID-I) iii. Beck Depression Inventory iv. Word Memory Test	Generally, well-recovered patients who underwent a small trauma more than 5 years ago still experience long-term cognitive and emotional sequelae that are important for their day-to-day social and professional lives.
	Fortier-Lebel et al. (75)	To investigate the impact of a single and acute mild TBI on episodic memory and structural cerebral changes.	Experiment 1: A comparison study was conducted to evaluate verbal episodic memory using a word recall test (*n* = 52 mild TBI patients, 54 healthy controls). Experiment 2: MRI analysis was conducted to evaluate the hippocampus volume of several participants from Experiment 1 (*n* = 20 mild TBI patients, 20 controls), and memory performance scores to hippocampal volume were correlated.	An acute single mild TBI episode is associated with both episodic memory alteration and reduced volume of the hippocampus in the acute phase.
	Taing et al. (46)	To study the category-specific impairment in the episodic memory of TBI patients.	fMRI-based task (*n*= 43 moderate-to-severe TBI patients, 38 healthy controls). During fMRI, participants were shown several pictures (face, scene, and animal) and had to properly identify the stimuli across two presentation runs in an out-of-scanner task.	The TBI group showed impaired memory for people and scenes, but not animals, indicating a category-specific impairment in TBI.
Priming	Watt et al. (76)	To study the performance of implicit and explicit memory in severe TBI patients.	An experiment involving both the implicit (word-stem completion test) and explicit (cued-recall test) tasks was conducted (*n* = 12 severe TBI patients, 12 healthy controls). The same experiment was repeated 3 months later.	The word-stem completion performance of the TBI patients was significantly affected by the unavailability of extra attentional resources, which were not retained due to TBI.
	Vakil and Oded (77)	To investigate the memory performance of the CI patients in the learning and relearning tasks.	20 CI patients and 20 controls (aged 20–42 years old) were tested with three different memory tasks: cued recall, word stem completion (WSC), and saving.	CI patients exhibited impairment in explicit memory, although the learning rate is preserved. Implicit memory is preserved in CI patients only when based on the reactivation of preexisting knowledge, but not when dependent on forming new associations.
Procedural	Ewert et al. (78)	To investigate the performance of procedural memory following a post-traumatic amnesia event.	A comparison study between 16 amnesic CI victims and 16 controls was conducted. The procedural learning exercises involved mirror reading, mazes and a pursuit rotor job that required following a rotating object.	CI patients showed impaired memory for word lists and recent events (declarative memory), as well as a deficit in the acquisition of skills (procedural memory).
	Shum et al. (79)	To investigate the preservation of implicit memory following a severe TBI event.	A comparison study between 16 patients with TBI and 16 controls was done using two explicit memory measures (graphemic-cued recall and semantic-cued recall) and two implicit memory tasks (word-fragment completion and general knowledge).	TBI patients performed noticeably worse on the explicit memory tasks but not on implicit memory tasks compared to control, indicating that the implicit memory is preserved following TBI.
	Rigon et al. (80)	To compare the procedural memory performance of TBI patients with control.	A comparison study between 36 TBI patients and 40 controls was conducted. The rotary pursuit task was administered. The rate of learning and mastery of the skill were compared.	TBI patients exhibit comparable performance on the rate of procedural learning compared to healthy controls. However, there were significant individual variations in both groups, suggesting that some people might not benefit from therapeutic strategies that make use of a healthy procedural memory system.
Learning	Mutter et al. (81)	To assess the serial learning pattern after the TBI event.	Nonverbal serial pattern learning in patients with TBI was examined using a serial reaction time task (*n* = 12 TBI patients, 12 healthy controls).	Serial pattern learning and memory are not significantly affected by mild to moderate TBI.
	Vakil et al. (82)	To study the skill learning performance in severe CI patients.	Serial reaction time (SRT) task (*n* = 20 CI patients, 20 controls).	The CI group performed worse on the explicit measure of sequence learning. On one of the implicit measures of sequence learning, general response time learning, the groups did not differ. However, when it came to remembering the precise sequence that was repeated in the SRT exercise, the control group outperformed the CI group.
	Schwizer Ashkenazi et al. (83)	To investigate the effect of TBI on implicit sequence learning (ISL).	The ocular version of the serial reaction time (O-SRT) task was used to measure ISL (*n* = 26 TBI patients, 28 healthy controls).	The TBI group, in contrast to controls, was unable to mobilise their comparatively unharmed spatial WM capacity to support their ISL performance.

**Table 3:** VR-based cognitive rehabilitation therapy for memory following TBI

Research & Year	Method	Sample size	Memory assessment tools used	Outcome	Limitation
De Luca et al. & 2022 (99)	Subjects are divided into VR and conventional based cognitive therapy. Virtual Reality Rehabilitation System (VRRS-Evo) was used for VR therapy. Both control and treatment subjects underwent rehabilitation therapy three sessions per week for 8 weeks	30 subjects, divided into VR-based therapy (*n* = 15) and conventional therapy (*n* = 15)	Montreal Cognitive Assessment (MoCA).	Experimental intragroup analysis exhibited significant differences in the MoCA pre and post-test (*P* < 0.0006), and intergroup analysis also showed significant differences among the VR-treatment group and the conventional control group (*P* < 0.02)	Small sample size, allocation procedures were not concealed, and short-term follow-up
Yip and Man & 2013 (100)	A virtual reality-based prospective memory training programme was conducted using a non-immersive form of VR. Subjects were randomised to receive either VR or conventional-based cognitive therapy, where pre- and post-test results were compared. Cognitive therapy was conducted in 12 sessions. Training contents consisted of daily activities encountered by subjects	37 subjects, divided into VR-based therapy (*n* = 19) and conventional therapy (*n* = 18)	Behavioural checklist of prospective memory (PM) task in a real environment, The Cambridge Prospective Memory Test-Chinese version (CAMPROMT-CV)	The VR-based within-group analysis showed significant results in the CAMPROMT-CV scores pre and post-test (*P* < 0.05) The VR group and control group showed significant differences in real-life behavioural PM tests (*P* < 0.05), indicating better performance of prospective memory in the VR group compared to the control	The small sample size, short training period and training content were fixed in terms of the difficulty schedule, which might affect the subject’s performance. Non-individualised training programmes might not be suitable for some subjects
Caglio et al. & 2012 (101)	VR rehabilitation training employed navigational tasks, consisting of 3 sessions each week (90 min per session) for 5 weeks. The patient was evaluated before and after training using standardised neuropsychological assessments. He was retested 2 months later, and once again after 1 year	One 24-year-old male patient with TBI (maximum GCS score of 5) accompanied with memory deficit	Corsi Block-Tapping Test, Corsi’s Supraspan Test, Backward digit span, Alzheimer’s Disease Assessment Scale (ADAS), The Rivermead Behavioural Memory Test (RBMT), functional neuroimaging assessment	The patient exhibited memory improvement after VR training, as well as in follow-up studies. Functional neuroimaging data also indicated that the training enhanced activation in the hippocampal and parahippocampal brain regions. Both psychometric tests and neuroimaging findings indicated that VR-based memory training has the potential to enhance memory in TBI patients	Only one patient
Gamito et al. & 2011 (102)	All subjects underwent VR therapy, consisting of 10 online VR sessions (around 5 min per session). The VR platform consisted of a small town populated with digital robots (bots). Patients must conduct several tasks, including working memory tasks. Memory assessments were conducted pre-, during and post-VR sessions to assess memory performance	20 male subjects, divided into VR-based therapy (*n* = 19) and conventional therapy (*n* = 18)	Paced Auditory Serial Addition Task (PASAT)	Non-parametric pairwise comparison analysis revealed that there is a significant increase in the percentage of correct responses between pre- and during VR assessment (Trial 1 = *P* < 0.001, Trial 2 = *P* < 0.05) as well as during and post-VR assessment (Trial 1 = *P* < 0.001, Trial 2 = *P* < 0.05), exhibiting improvement in the working memory of the TBI patients	No control group, a small sample size, and a short therapy time
